# Changes in fluconazole pharmacokinetics can impact on antifungal effectiveness in critically ill burn patients: a Pharmacokinetic-Pharmacodynamic (PK/PD) approach

**DOI:** 10.1016/j.clinsp.2024.100491

**Published:** 2024-09-23

**Authors:** Victor Kaneko Matsuno, Edvaldo Vieira de Campos, Elson Mendes da Silva Junior, João Manoel da Silva Junior, David de Souza Gomez, Silvia Regina Cavani Jorge Santos

**Affiliations:** Faculdade de Ciências Farmacêuticas, Universidade de São Paulo, São Paulo, SP, Brazil

**Keywords:** Fluconazole, Pharmacokinetics, Candidiasis, Burn, Critical care

## Abstract

**Objectives:**

The Fluconazole pharmacokinetic-pharmacodynamic relationship was investigated in a few clinical settings and only limited studies regarding burned patients are available. Thus, the authors aimed to investigate fluconazole pharmacokinetics changes and its impact on antifungal therapy coverage against dose-dependent *Candida spp.* applying the PK/PD approach in critically ill severely burned patients.

**Methods:**

Fluconazole was administered as a one-hour intravenous infusion of 200 mg q12h. Doses were increased according to the coverage based on the PK/PD approach. Blood samples were collected at the end of the infusion (1^st^ hour), two hours after (3^rd^ hour), and before the next dose (12^th^ or 24^th^ hour). Serum concentrations were obtained by HPLC-UV. Pharmacokinetic parameters were estimated by noncompartmental analysis and compared with data described in healthy subjects. The effectiveness predictive index was based on the AUC^ss^_0-24h_/MIC ratio, with a target above 25.

**Results:**

Every pharmacokinetic parameter was reduced throughout all three sets of the study. Compared to healthy subjects, the volume of distribution was decreased about 3‒7 times, biological half-life was 2‒3 times shorter and total body clearance was slightly altered but statistically significant. Both half-life and total body clearance were correlated to the volume of distribution. Consequently, an increase in fluconazole daily dose was necessary to improve empiric coverage.

**Conclusions:**

Fluconazole pharmacokinetics is altered in critically ill severely burned patients, mainly related to the volume of distribution. Doses higher than usual may be necessary to reach the PK/PD target and guarantee antifungal coverage against dose-dependent *Candida spp.* up to MIC 32 mg/L.

## Highlights

• Fluconazole pharmacokinetics is significantly altered in critically ill burn patients.
• Half-life and total body clearance were correlated to volume of distribution decrease.
• High-dose fluconazole may be necessary to guarantee coverage against *Candida glabrata*.

## Introduction

Burn wounds are associated with high morbimortality and therefore have a markedly impact on economics and healthcare systems. The mortality rate is estimated at around 180,000 cases per year worldwide, with higher prevalence in developing countries since socioeconomic status is the main factor associated with poor outcomes.^1^^,^^2^

Burned patients are more susceptible to fungal infections as a result of skin barrier disruption and immune dysregulation induced by the burn and the tissue repair process.^3^, ^4^, ^5^

*Candida spp.* represents about 80% of nosocomial fungal infections and it is estimated around 2.5 cases per 1000 admissions along with mortality rates above 50% in critical care units. In addition, invasive fungal infection diagnosis can be challenging because of confounder factors and prolonged time to culture positivity.^6^, ^7^, ^8^

Sepsis-related mortality increases with partial effectiveness or inefficacy of the antimicrobial therapy, which depends on the interaction between the antimicrobial drug, pathogen, and host. In general, it can be outlined as the patient's clinical status and the antimicrobial therapy Pharmacokinetic-Pharmacodynamic (PK/PD) relationship.^9^, ^10^, ^11^

Systemic inflammatory and metabolic responses to severe burns affect drug pharmacokinetics significantly. Haemodynamic impairment and shock, edema, and protein synthesis shift, among others, can lead to several changes in pharmacokinetic parameters.^12^^,^^13^

Few fluconazole studies regarding PK/PD approach are available, and even fewer considering severely burned patients. Hence, it is expected that fluconazole conventional doses may not guarantee appropriate coverage during septic shock in extensively burned patients, in the same way, that it has already been shown to other hydrophilic antimicrobial agents and patient populations.^14^, ^15^, ^16^, ^17^, ^18^

The authors hypothesize that critically ill burned patients present altered pharmacokinetics which may benefit from higher doses of fluconazole therapy to achieve adequate PK/PD effectiveness prediction index. Thus, the aim of this study was to investigate fluconazole pharmacokinetics in burned patients and evaluate its impact on PK/PD target attainment against *Candida spp.* with dose-dependent susceptibility such as *C. glabrata* (MIC 16‒32 mg/L).

## Materials and methods

### Study design

A prospective cohort investigation was conducted in a tertiary public hospital's 16-beds burn intensive care unit. Eight adult patients (> 18-years) severely burned (total body surface area burned > 15%), with documented *C. glabrata* infection, in septic shock requiring vasopressor therapy and receiving intravenous fluconazole as antifungal therapy for more than 72h were enrolled. Exclusion criteria at baseline were severe neutropenia (< 500 mm^3^), renal impairment (creatinine clearance < 50 mL/min, estimated by Cockcroft-Gault formula) or in renal replacement therapy, pregnant and lactating women, or patients with hypersensibility to fluconazole or any drug formulation excipient.

### Fluconazole administration and therapeutic drug monitoring

Fluconazole was initiated at the recommended empirical dose, 200 mg twice daily (set 1), followed by 400 mg once daily (set 2), and afterward increased to 400 mg twice daily (set 3) for optimization of the antifungal therapy. Every dose was administered as a one-hour Intravenous (IV) infusion.

Blood samples of 2 mL were collected at the steady state (after at least 72h of fluconazole therapy), at the end of the one-hour infusion (1^st^ hour), two hours after the end of the infusion (3^rd^ hour) and before the next dose (12^th^ or 24^th^ hour depending on dose regimen).

Bioanalytical procedures including purification of blood samples followed by fluconazole extraction and quantification method were developed and validated in a previous study.^19^ Serum samples were obtained by centrifugation of the blood at 2800g at room temperature for 20 min. Fluconazole was extracted from serum by liquid-liquid chromatography with dichloromethane in an alkaline medium. After centrifugation (at 2800 g, 5°C), the aqueous phase was discarded, and the organic phase was cooled for decantation in an ice bath and then dried to residue under a nitrogen stream. Then the residue obtained after dichloromethane evaporation was redissolved in a mixture of acetonitrile and ultrapure water (6:4 v/v), transferred to an analytical microvial and inserted into the chromatograph autosampler rack, which also contained the calibration curve and internal quality controls. Quantification was performed by high-performance liquid chromatography with ultraviolet detectors set at 210 nm (Shimadzu LC10 ADvp, Kyoto, Japan). The chromatographic system was a Shimpack CN column of 150 × 6.0 mm, 5 μm (Shimadzu, Kyoto, Japan), and a binary mobile phase of acetonitrile and ultrapure water (6:4 v/v). A 15-min isocratic analytical run was performed to guarantee selectivity.

### Pharmacokinetic-pharmacodynamic approach

Fluconazole pharmacokinetics was performed by a noncompartmental data analysis. Pharmacokinetic parameters were estimated based on a one-compartment open model by first-order linear equations as described in Supplementary Table 1. The Area Under the Curve (AUC) was integrated by the trapezoidal rule.^20^

Fluconazole effectiveness predictive index was calculated based on the area under the curve of 24h (AUC^ss^_24h_) and the theoretical Minimal Inhibitory Concentration (MIC) for *Candida spp.* with dose-dependent susceptibility (MIC 16‒32 mg/L). An ASC^ss^_0-24h_/MIC ratio above 25 was considered as the therapeutic target recommendation.^21^^,^^22^

To establish a reference range for each fluconazole pharmacokinetic parameter, data from healthy volunteers was extracted from the literature^23^, ^24^, ^25^, ^26^, ^27^, ^28^, ^29^ and a meta-analysis was performed considering mean values, variance/standard deviation, and sample size. Combined healthy volunteers' half-life, Volume of distribution (Vd), and total body Clearance (CL_T_) are 27.5 ± 9.3h, 48.70 ± 11.05 L and 1.18 ± 0.30 L/h, respectively, as described in Supplementary Table 2.

### Statistical analysis

Pharmacokinetic data from the burned patients were expressed as median (interquartile range) and analyzed by GraphPad Prism for Windows v9.1.1, GraphPad Software Inc. (San Diego, California, USA) using a non-parametric comparative method for paired samples (Wilcoxon signed-rank test) with a significance level of 5% (p < 0.05). Data correlation was performed by Pearson linear correlation coefficient, considering a significance level of 5% (p < 0.05) and a 95% Confidence Interval.

## Ethics

Protocol was registered and approved by the hospital ethics committee (CAEE 07525118.3.0000.0068). Every patient was presented with written informed consent. The signature of the patient or legal guardian was collected at the end of the orientation. This study was also conducted in accordance with the World Medical Association Declaration of Helsinki Ethical Principles.

## Results

Baseline patient characteristics are presented in Table 1. Eight critically ill burned patients (4 female and 4 male) in septic shock with invasive candidiasis were enrolled in the study.

**Table 1:** Descriptive characteristics of critically ill severely burned patients.

**Demographic characteristics**	
Female/Male	4/4
Age (years)	35.5 (31.2‒42.8)
TBW (kg)	70.0 (61.2‒73.8)
IBW (kg)	62.5 (52.0‒70.0)
BSA^a^ (m^2^)	1.81 (1.66‒1.87)
BMI^a^ (kg/m^2^)	24.2 (23.5‒25.9)
Baseline/Admission characteristics	
TBSA burned (%)	46.0 (36.0‒53.8)
Mechanical ventilation	7/8
SAPS-3*	61.0 (55.0‒67.8)
Urea (mg/dL)	29.0 (28.0‒31.8)
SCr (mg/dL)	0.83 (0.71‒1.04)
CL_SCr_^a^ (mL/min)	106.7 (94.7‒132.8)
CRP (mg/L)	3.4 (2.4‒112.8)
Lactate (mg/dL)	26.5 (19.0‒37.5)
WBC (1,000/mm^3^)	13.02 (10.78‒18.43)
Neutrophils (1,000/mm^3^)	9.84 (6.18‒15.80)
Platelets (1,000/mm^3^)	274.0 (227.3‒361.8)
Clinical/surgical characteristics during hospital stay	
Vasoactive drug	8/8
Surgery	9.0 (4.2‒14.2)
Escharotomy/grafting	5.5 (4.0‒8.0)
Amputation and revision surgery	1.5 (0.0‒6.0)
Microbiological cure	8/8
ICU LOS (days)	49.0 (37.2‒93.0)
Hospital LOS (days)	57.0 (37.2‒153.0)
Death	4/8

Values are presented as median (interquartile range) or as absolute count.

BMI, Body Mass Index; BSA, Body Surface Area; CL_SCr_, Creatinine clearance; CRP, C-Reactive Protein; IBW, Ideal Body Weight; ICU, Intensive Care Unit; LOS, Length of Stay; SAPS-3*, Simplified Acute Physiology Score (for Latin America); SCr, Serum Creatinine; TBSA, Total Body Surface Area; TBW, Total Body Weight; WBC, White Blood Cells.

The median age and weight were 36 years old and 70 kg, respectively. Only one of the patients was obese (body mass index of 35.3 kg/m^2^), but the dose regimen was estimated based on the ideal body weight for all of them. At admission, patients had a median SAPS-3* of 61 and a median Total Body Surface Area (TBSA) burned of 46%. Nearly all patients had inhalation injuries (7/8), requiring mechanical ventilation for airway protection.

All of the patients required vasoactive drugs due to hypotension and septic shock, maintaining it throughout all three treatment regimen sets. Several surgical procedures were performed, such as debridement, escharotomy, grafting, amputation, and revision surgery with a median of nine surgeries per patient. Patients were hospitalized for a median of 57 days with a median Length of Stay (LOS) in the Intensive Care Unit (ICU) of 49 days. All patients achieved microbiological cure from invasive candidiasis in spite of the death of half of them.

Patients received a median fluconazole dose of 5.71 mg/kg/day initially (set 1 and 2), then increased to 11.43 mg/kg/day in set 3 (based on ideal body weight). Fluconazole AUC^ss^_0-24h_ was directly proportional to the daily dose (Pearson *r* = 0.7685, p < 0.0001). Effectiveness predictive index of AUC^ss^_0-24h_/MIC above at least 25 was achieved in all patients considering a MIC of 16 mg/L. In contrast, for a MIC of 32 mg/L, the target was reached only when the fluconazole dose was optimized (800 mg/day, set 3).

**Figure 1:**
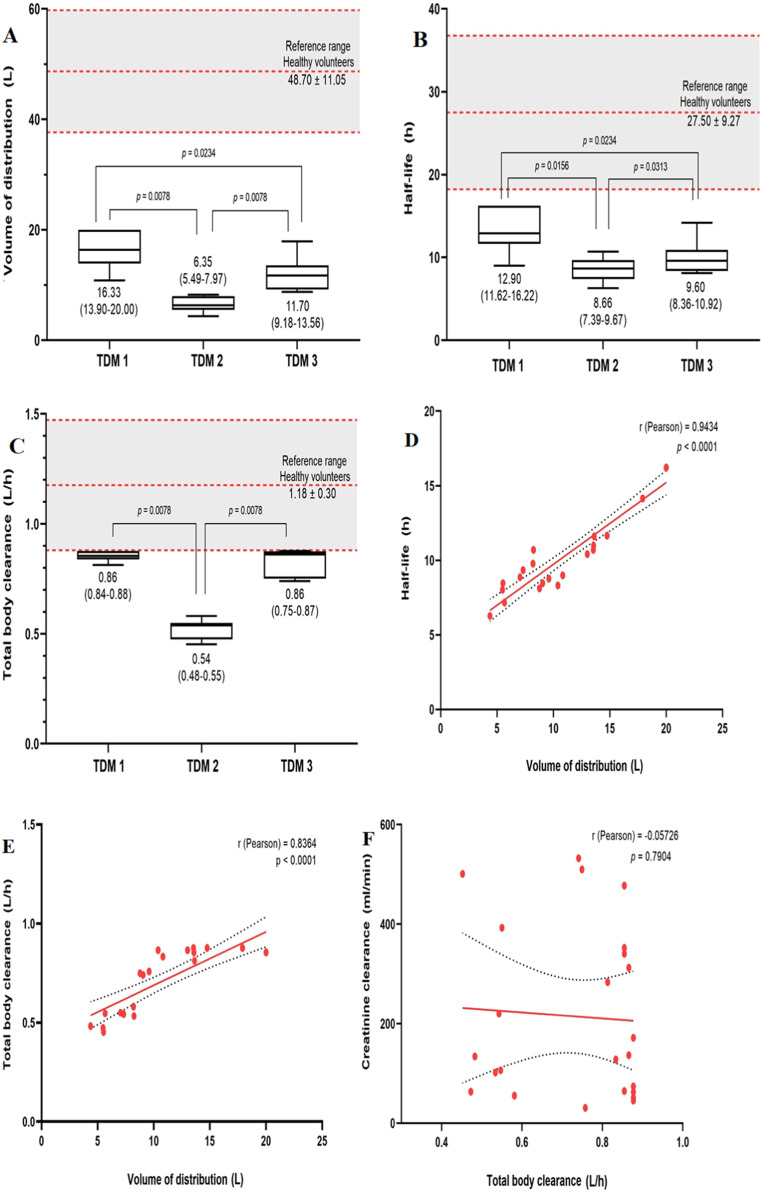
Fluconazole pharmacokinetic parameters in critically ill severely burned patients. (A) Volume of distribution, (B) Half-life, (C) Total body clearance, (D) Linear correlation of volume of distribution and half-life, (E) Linear correlation of volume of distribution and total body clearance, (F) Linear correlation of total body clearance and creatinine clearance. The reference range is presented as mean ± standard deviation. Data from this study is presented as median (interquartile range). Statistics: GraphPad Prism 9.1.1 (software); Wilcoxon signed-ranks test, a significance level of p < 0.05. TDM, Therapeutic Drug Monitoring.

**Figure 2:**
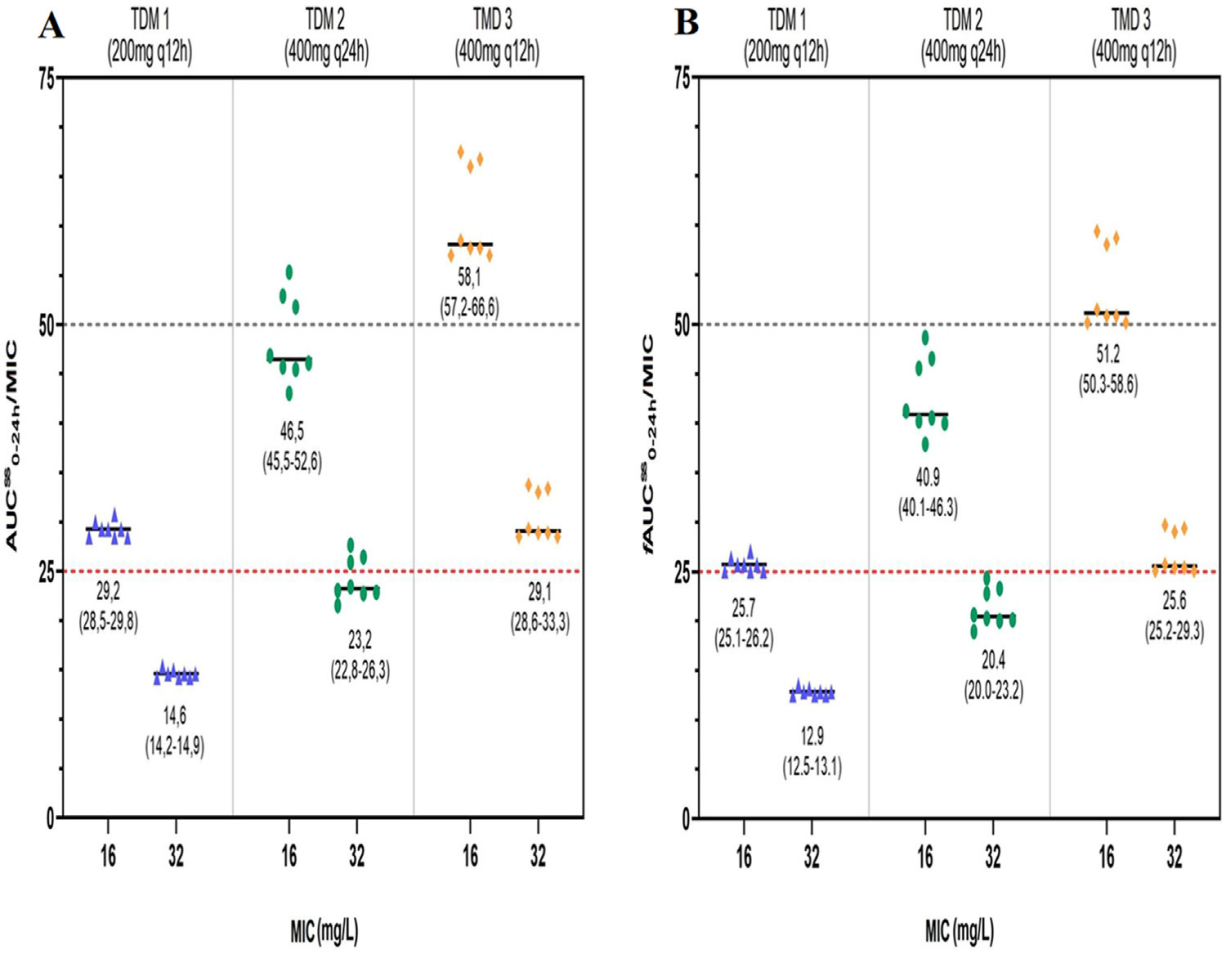
Fluconazole PK/PD target attainment in the critically ill severely burned patients. (A) AUC^ss^_0-24h_/MIC. (B) *f*AUC^ss^_0-24h_/MIC. Data from this study is presented as median (interquartile range). AUC^ss^_0-24h_, Area Under the Concentration-time curve of 24h; *f*AUC^ss^_0-24h_, Area Under the free Concentration-time curve of 24h; MIC, Minimum Inhibitory Concentration; TDM, Therapeutic Drug Monitoring.

Fluconazole pharmacokinetic parameters and data correlations are presented in Figure 1. Every pharmacokinetic parameter was reduced in the burned patients compared with data described in healthy subjects, as shown in Figures 1A, 1B and 1C. Half-life reduction was proportionally related to Vd in all regimen sets (Pearson *r* = 0.9434, p < 0.001, Fig. 1D). Whilst CL_T_ was lowered in a smaller magnitude and appears to be partially proportional to Vd (Pearson *r* = 0.8364, p < 0.001, Fig. 1E). However, there was no correlation between estimated Creatinine Clearance (CL_SCr_) and fluconazole CL_T_ (p = 0.7904, Figs. 1F and 2).

## Discussion

It is known that critically ill burned patients are more susceptible to invasive candidiasis and often experience changes in drug pharmacokinetics which can impact antifungal coverage and clinical outcome.^4^^,^^5^ However, there are insufficient fluconazole Pharmacokinetic-Pharmacodynamic (PK/PD) studies to establish a universally accepted effectiveness predictor index. Only two studies included burned patients.^30^^,^^31^

Boucher and colleagues^30^ investigated fluconazole pharmacokinetics in ten patients with second and third-degree burns (mean TBSA burned 50±24%) who received 400 mg daily intravenous infusions. They described a slightly reduced half-life (25.8±6.1 h) and moderately augmented Vd and CL_T_ (56±11 L and 1.65±0.40 L/h, respectively), which were opposed to this study. However, some significant differences can be acknowledged, for instance, patients with hemodynamic instability and vasopressor requirement were excluded.

Furthermore, Han and colleagues^31^ reported a pharmacokinetic model analysis of 60 burn patients who received 100 to 400 mg per day of fluconazole as a 10 to 40-min intravenous infusion. In spite of the fact that they do not describe every pharmacokinetic parameter, the data presented in the study implies that pharmacokinetic parameters were close to those reported in healthy volunteers. Again, the population enrolled did not include hemodynamically unstable patients. And, although APACHE II and SAPS-3* are not directly comparable, the reported mean APACHE II of 7.6 suggests a lower clinical severity and mortality risk than the patients here presented. In addition, the male proportion was considerably higher (50M/10F), which can impact population pharmacokinetics, since it has been already reported sex-related differences in half-life and Vd.^32^

Such differences can also be explained by other factors, such as the stage of the burn. Both studies described patients mainly in the early phase from the injury (< 30 days), whilst the patients started fluconazole around day 26 (median, range 14‒40 days). Therefore, to our knowledge, it is the first fluconazole pharmacokinetic description in critically ill severely burned patients in both the early and late phase of the burn, and also performing drug therapeutic monitoring in a three-set longitudinal analysis.

Fluconazole pharmacokinetic parameters were reduced throughout all three sets with a marked impact on Vd. The three to seven-fold reduction of Vd could be explained by the extensive burn lesions since prolonged half-life and high Vd of fluconazole are attributed to accumulation in epithelial tissue. It has been described that dermal-epidermal and stratum corneum concentrations are higher than serum concentration reaching up to 90-fold during prolonged fluconazole therapy.^33^, ^34^, ^35^, ^36^

Additionally, stratum corneum concentration drops as soon as fluconazole is discontinued, however, dermal-epidermal concentration can remain stable for four to five days after the end of antifungal therapy, and even after 10 days fluconazole can be detected in considerable concentrations as fluconazole clearance from corneum stratum is about two to three times slower than plasma elimination half-life.^36^, ^37^, ^38^

On the other hand, the influence of Vd over CL_T_ does not justify CL_T_ decrease as it would be expected to be unaltered or even increased due to reduction of accumulation which would bring the serum concentration up and, thus, fluconazole would be more favorably cleared by the kidneys.

The reduction of CL_T_ might reflect renal impairment associated with sepsis and hemodynamic instability. Besides that, protein-bound shift is well described in several clinical settings, including sepsis, cancer, chronic kidney disease, inflammatory conditions, and tissue injury/repairment processes.^39^, ^40^, ^41^, ^42^, ^43^, ^44^

Alpha-1-acid glycoprotein is one of the acute-phase proteins that are upregulated during burn-induced tissue damage. It is associated with immunomodulation, fibroblast protection, and transport of inflammatory substances, as well as several drugs.^39^^,^^40^^,^^45^, ^46^, ^47^, ^48^

Burn extent and tissue recovery time appear to be correlated to alpha-1-acid glycoprotein levels.^49^^,^^50^ Production starts rapidly in the initial 12 to 24 hours after the burn injury and can reach up to four-fold concentration in the next two weeks. After tissue recovery, alpha-1-acid glycoprotein concentration gradually decreases with a half-life of around two to five days but can remain significantly augmented over 20 days up to six weeks.^45^^,^^48^^,^^49^^,^^51^^,^^52^

Although there are no studies that evaluate fluconazole protein binding in burned patients, there is a significant correlation between alpha-1-acid glycoprotein serum concentration and fluconazole protein binding in chronic scenarios with an almost two-fold rise.^41^^,^^42^

Moreover, it would be likely to be even greater in burned patients since acute inflammatory conditions are associated with up to six times higher alpha-1-acid glycoprotein levels^48^ and, thus, its overexpression during burn-induced tissue injury/repairment and septic shock could explain the observed CL_T_ reduction.

As exploratory data, it was noticed that every patient presented hypoalbuminemia during all three sets (mean 1.7 ± 0.6 g/dL) with a significant difference between the first and last measured serum albumin (2.8 ± 0.5 vs. 1.5 ± 0.4 g/dL, p < 0.0001), which is compatible to the expected shift on protein production. However, serum alpha-1-acid glycoprotein was not routinely monitored.

The authors estimated *f*AUC^ss^_0-24h_/MIC assuming a 12% fluconazole protein binding as described in healthy subjects. Revising fluconazole coverage based on a higher protein binding of 22.9% as reported by Arredondo and colleagues,^41^^,^^42^ *f*AUC^ss^_0-24h_/MIC > 25 would be achieved only when MIC = 8 mg/L in the first set and when MIC = 32 mg/L only three patients of the last set would be covered.

There are some limitations regarding this study. This is a single-center experience with a small sample size and does not have a comparator group, therefore caution may be necessary when generalizing these results. The present study enrolled only enough patients to detect pharmacokinetics differences but not to elucidate those pharmacokinetic change mechanisms. Also, it was not the purpose of this study to assess clinical outcomes or compare treatment efficacy among those who reached or did not the therapeutic target. Therefore, further and larger studies are necessary to confirm these hypotheses and to better assess clinical outcomes and endpoints.

In addition, only patients without renal impairment were enrolled and Therapeutic Drug Monitoring (TDM) was performed for all patients, which reduced toxicological concerning. In most clinical settings TDM is not feasible because there is not a commercial kit available for fluconazole TDM. Thus, kidney and liver function, as well as drug interactions, should be monitored during high-dose fluconazole treatment since it is expected to have a higher risk of kidney and liver impairment.

Finally, data reported by this study can contribute to better clinical decisions regarding fluconazole antifungal therapy in critically ill burned patients.

## Conclusions

This is the first longitudinal fluconazole pharmacokinetic characterization study in critically ill severely burned patients at both the early and late phase of the burn and performed by PK/PD approach. Pharmacokinetic parameters differ significantly from healthy volunteers and these changes impact directly on antifungal therapy effectiveness predictive index. Consequently, higher than standard fluconazole doses are necessary in these clinical settings to guarantee empirical coverage against *Candida spp.* up to MIC 32 mg/L. Considering that most bedside and clinical settings where fluconazole therapeutic drug monitoring is not feasible, close monitoring of kidney and liver function should be granted before and throughout high-dose fluconazole treatment as it is expected to have a higher risk for toxicity.

## Authors’ contributions

Victor Kaneko Matsuno: Conceptualization; Data curation; Formal analysis; Investigation; Methodology; Visualization; Writing - original draft; Writing - review & editing.

Edvaldo Vieira de Campos: Investigation; supervision; project administration.

Elson Mendes da Silva Junior: Investigation; supervision; project administration.

João Manoel da Silva Junior: Investigation; supervision; project administration.

David de Souza Gomez: Conceptualization; development and design, investigation; writing-review & editing; supervision; project administration.

Silvia Regina Cavani Jorge Santos: Conceptualization; Data curation; Formal analysis; Investigation; Methodology; Project administration; Supervision; Validation; Visualization; Writing - review & editing.

## Funding

Master's degree scholarship (personal) ‒ CNPq / Mestrado ‒ GM (133851/2019-2) ‒ Victor Kaneko Matsuno.

Research Projects ‒ Thematic Grants (institutional) ‒ FAPESP (2018/05616-3) / “Farmacocinética clínica em doenças infecciosas” ‒ https://bv.fapesp.br/pt/auxilios/102788/farmacocinetica-clinica-em-doencas-infecciosas/.

## Declaration of competing interest

The authors declare no conflicts of interest.

## Appendix Supplementary materials

Image, application 1
