# Supplementary material for: Changes in fluconazole pharmacokinetics can impact on antifungal effectiveness in critically ill burn patients: a Pharmacokinetic-Pharmacodynamic (PK/PD) approach
Source: Clinics (Sao Paulo). 2024 Sep 23;79:100491. doi: 10.1016/j.clinsp.2024.100491 (PMC11462178; doi:10.1016/j.clinsp.2024.100491)
Supplement: Supplementary file 1 [file mmc1.docx]

**CLINICS-D-24-00861_Supplementary Material**

**Supplementary Table 1** Intermittent multiple infusions pharmacokinetics equations.

| **Description** | **Equation** | **Unit** |
| --- | --- | --- |
| Elimination rate constant | $k_{el} = \frac{lnC_{1} - ln C_{2}}{t_{2} -t_{1}}$ | NA |
| Elimination half-life | $t_{1/2} = \frac{0.693}{k_{el}}$ | h |
| Maximum concentration | $C_{max}=\frac{C_{1}}{e^{-k_{el} {\times(t}_{1}- Tinf)}}$ | mg/L |
| Minimum concentration | $C_{min}=C_{max} \times e^{-kel \times(tau-Tinf)}$ | mg/L |
| Volume of distribution | ${Vd}=\frac{{CL}_{T}}{k_{el}}$ | L |
| Total body clearance | ${CL}_{T}=\frac{Dose}{{AUC}}$ | L/h |
| Area under the concentration-time curve | ${AUC}_{0-t}=t \times\frac{{(C}_{max}+ C_{min})}{2}$ | mg.h/L |

AUC, Area Under the Concentration-time curve; C_1_, Concentration at time t_1_; C_2_, Concentration at time t_2_; C_min_, maximum (or peak) concentration; C_max_, Minimum (or trough) concentration; CL_T_, Total body clearance; h, hour; k_el_, Elimination rate constant; L, Liters; mg, milligrams; NA, Not Applicable; Tinf, Infusion time; t_1/2_, Elimination half-life; t_1_, Time at which C_1_ was collected; t_2_, Time at which C_2_ was collected; tau, Time between doses (dosing interval); Vd, Volume of distribution.

Adapted from Dipiro JT, Spruill WJ, Wade WE, Blouin RA, Pruemer JM. Concepts in Clinical Pharmacokinetics. 5^th^ ed. Bethesda, Maryland, USA: American Society of Health-System Pharmacists; 2010; NonCompartmental Data Analysis ‒ PK Solutions v.2.0 (software).

**Supplementary Table 2** Fluconazole pharmacokinetics in healthy subjects.

| **Reference** | **Population** | **Regimen** | **t_1/2_ (h)** | **CL_T_ (L/h)** | **Vd (L)** |
| --- | --- | --- | --- | --- | --- |
| Humphrey et al. (1985) | Healthy men (n = 4) | Single dose 1 mg/kg (63‒70 mg) PO | 22 ± 3.5 | 1.68 ± 0.17ª | 49.0 ± 4.2^a^ |
| Foulds et al. (1988) | Healthy men (n = 10) | 50‒100mg IV | 29.63 ± 2.18 | 1.34 ± 0.052 | 47.3 ± 6.6 |
|  |  | Tinf 30 min |  |  |  |
| Shiba et al. (1990) | Healthy subjects (n = 8) | 25‒50 mg IV | 33.3 ± 2.7^b^ | ND | 51.68 ± 7.73^b^ |
|  |  | Tinf ND |  |  |  |
| Toon et al. (1990) | Healthy subjects (n = 5) | Single dose 50 mg PO | 31.2 ± 4.59 | 1.43 ± 0.46 | 63.7 ± 16.1^c^ |
| Ripa et al. (1993) | Healthy subjects (n = 6) | Single dose 100 mg IV | 29.73 ± 8.05 | 1.26 ± 0.30 | 52.16 ± 9.83 |
|  |  | Tinf. 30 min |  |  |  |
| Yeats et al. (1994) | Healthy subjects (n = 10) | Single dose 100 mg IV | 35 ± 3 | 1.38 ± 0.24 | 64.86 ± 3.45^d^ |
|  |  | Tinf 20 min |  |  |  |
| Guo et al. (2010) | Healthy subjects (n = 60) | Single dose 200 mg PO | 31.10 ± 5.26^e^ | 1.06 ± 0.26^e^ | 47.16 ± 11.77^e^ |
| Combined data | (n = 103) | ‒ | 27.50 ± 9.27 | 1.18 ± 0.30 | 48.70 ± 11.05 |

CL_T_, Total body Clearance; h, hour; IV, Intravenous; L, Liters; mg, milligrams; ND, Not Described; PO, Orally; Tinf, Infusion Time; t_1/2_, Elimination half-life; Vd, Volume of distribution.

Notes:

^a^ Based on a 70 kg subject (population age range: 63‒70 kg).

^b^ Based on the partially available published data.

^c^ Based on a 70 kg subject.

^d^ Based on the mean weight of 69 kg.

^e^ Based on the five subpopulations.

Statistics: RevMan calculator (Cochrane Training).
